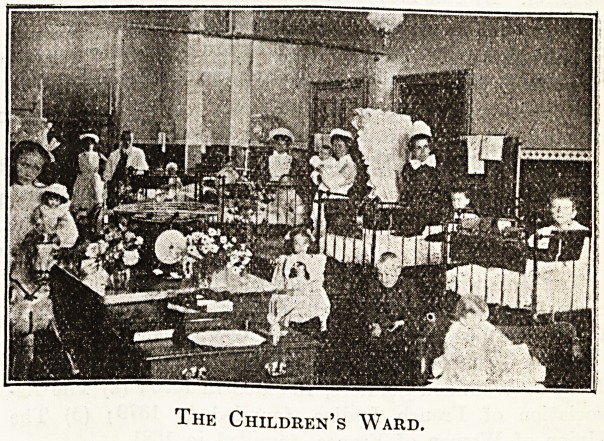# The Hospital Spirit in Torquay

**Published:** 1912-12-14

**Authors:** 


### THE HOSPITAL SPIRIT IN TORQUAY.

### Mr. H. J. Packe on the Progress of the Torbay Hospital.

The Southern Counties, as is well known, offer a very different problem to hospital managers from that presented by the factory districts of the Midlands and the North; but though the voluntary system in the South naturally does not express itself by the raising of elaborate organisations among workpeople, nor by the growth of huge Hospital Saturday Funds, it has as deeply marked a current of life, as Mr. H. J. Packe explained to our Commissioner

The Torbay Hospital from the Garden.

who visited the Torbay Hospital, Torquay, to gain some idea of its progress.

"The turning-point in our history," Mr. Packe began, "really occurred in 1893, when the hospital was re-opened in its present fine position at the top of Union Street. Founded in 1844 as an 'infirmary and dispensary,' in-patients were not admitted till 1848, and the first stone of the actual hospital was not laid till 1850. A wing for fever patients was built in 1851, and, thanks to an anonymous donor, a second wing was added in 1878. Thus, briefly, matters remained till 1892, when the gift of £7,000 made it possible to undertake the reconstruction of the institution. Now the close connection of the hospital with the town cannot be better illustrated than by mentioning the outcry which was raised when it became known that the board did not like the site, and wished to move to one in a less central and more adaptable position. Many of the public at once threatened to withdraw their subscriptions, and though the site is an oblong island with no possibility of extension, and so far inconvenient, it was decided that our subscribers' wishes must be agreed to, so the hospital remained. Now the efforts of nearly sixty years are being threatened by the Insurance Act, which is causing the gravest anxiety to all connected with the medical or institutional management of the hospital."

### The Effect of the Insurance Act.

"What have been its immediate effects?"

"So far, Hospital Saturday, which in the nature of things can never reach a huge sum in a seaside resort like Torquay, has decreased its yield, and we have had to postpone consideration of building a new $x$-ray department, which is badly needed, on account of the Act. In another small way the evil effect is indicated. For many years past I have visited the public-houses, and, thanks to the courtesy of the publicans, have raised in this way very useful sums of money. The shopkeepers, however, are now refusing to subscribe, and it has become a sad joke now that I have to repeat so often to those in the public-house, 'You can still afford to spend money here; cannot you still afford to subscribe to the hospital?' In another way the Act will seriously affect us. A few days ago I made a calculation and discovered that one-third of the in-patients then in the hospital would be insured persons under the Act. As regards the out-patients, we have no strict out-patient department, but the provident dispensary, out of which the hospital has grown, is continued, and large numbers of casualties come to the hospital. The proposed $x$-ray department illustrates our difficulty; are we to wait six months, or are we to spend capital, only perhaps to find that the doctors won't treat insured persons? If they refuse to treat insured persons unless these are paid for, are we justified in spending the money? Then, again, the younger medical men are much worried by the Act. Such men as the £300 a year club doctors, for instance. At present, too, the villa residents are refusing to subscribe, and it is no use to worry them; it can only be hoped that eventually they will return to their old allegiance. Hospital Saturday will, I am afraid, be seriously affected; some, indeed, believe that two-thirds of the fund will be lost. The present position is not so bad, for, though we have a debt of £300, the autumn quarter is generally the best."

### The Value of the Uniform System.

"Are the accounts kept on the Uniform System?"

"Yes, and I would not be without it for the world. The board required some persuasion at first, but in 1910 they were induced to adopt the system. In addition, however, to the books published by The Scientific Press for the working of the system, I keep also a ledger for the general treasurer's account, into which the various totals are transcribed. I have, however, one small criticism to make of the arrangement of the books for keeping the Uniform System. It is that the column at the extreme right-hand side of the page is too narrow to allow large totals to be written with comfort. Unless you have two sets of books, one large and one small, and ours is the

The Children's Ward.

small, the margin is too narrow. Speaking of accounts reminds me of a curious difficulty in which we were placed by a request from the late Mr. Lavers' Executors, who wanted the income derived from his legacy of £5,000 to be entered as a subscription. The result of acceding to the wish was that a subscriber criticised sharply the published accounts, on the ground that the capital account showed no increased revenue notwithstanding the Lavers legacy. Of course, the matter was easily explained, but it is an example of the difficulties which arise from testators who attach peculiar conditions to their bequests."

"I notice you have two Florence Nightingale Wards?"

"Yes, I think we may claim to be one of the first hospitals to recognise the value of her work, for these wards were named after her so long ago as 1873."

An Object-Lesson in Finance.

At this point Mr. Packe led the way over the hospital, pointing out, as we ascended, the new automatic electric lift. When we reached the woman's ward on the top floor he said:

"Interchange of experience is sometimes useful, and so you may be interested to hear how recently we raised enough money to re-bed the hospital. The sum needed for this purpose was £200, which the board were afraid would be a difficulty. It was decided, however, to place one of the existing old-fashioned beds with its straw palliasse on view, and to set beside it one of the latest patterns. These were shown to every visitor, with the result that in six weeks the money was raised. Another side of our finance is seen in this cot, which is maintained by the school children. Their interest in it is very great, and last year the sum that they subscribed reached £70."

"Have you an almoner?"

"No, for as I said, the provident dispensary here takes the place of an out-patient department. But the difficulty is got over in a way which is somewhat typical of the less highly organised but more personal system of hospitals in the South and in small towns everywhere. My long residence in Torquay—and I have held the post of secretary here for nearly twenty-five years—has made me personally acquainted with practically every family in the place. So I should know very quickly if there were any hanky-panky going on. It is curious that, though in my time the expenditure of the hospital has grown from £1,800 to £3,601, the debt has remained practically constant. It is not so long ago that we had a hand-power lift in place of the existing electric one. But four years ago Tuck's firm offered a prize for a post-card chain in favour of the hospital. By this means £250 was raised; the late matron, Miss Fortescue, and her night nurses used to put these in albums, and when the time-limit of the competition closed these were sent to London. It was then decided that this money should go to the lift. But even then our difficulties were not over. The electric light in the town was on the alternating system, with the result that the town's lights were liable to go out, and this led to objections, which were met only when a direct system for the purpose of supplying lifts and motors was added. Previously it had not extended beyond the town hall, which you passed a little way down the hill on the way to the hospital, and it had to be brought here. It is by design a very slow lift, as it was felt that this would add to the comfort of the patients."

The Needs of To-day.

"Then as to the future?"

"I have referred already to certain limits of the site. They can be illustrated by the fact that we badly need a new mortuary, viewing room, and private chapel. But there is not much room on which to build. But the site has other compensations. These did not come by chance. In 1887 it was suggested that the town should buy the land opposite in commemoration of the sixtieth year of the reign of the late Queen Victoria, and give it to the hospital, so that it would never be built over. This has been a fine thing for the hospital. Then, again, what has proved a very useful investment was made four years ago, when the house on the right of the hospital and its grounds were purchased. The sum of £1,100 was given for the freehold. The house has been let, and the grounds provide the one place on which extension is possible. There is a characteristic too about Torquay in general: people come, it is said, about eighty years of age and live as long as they like. Still some years our subscribers' list suffers heavily. Last year we had a record loss in this respect."

"What is your opinion of the present crisis?"

"I am a great supporter of the voluntary system. Apart altogether from the fact that any other would raise enormously the cost of hospital work. My life has been spent under the voluntary system, and I am convinced that nothing could ever replace the personal interest and personal service that work freely given alone affords. Sixteen years ago I had an opportunity of going elsewhere, and, though the prospect had material attractions, I found, as many others have found, that my affection for this hospital was stronger than any business consideration. Only the voluntary system can create such a feeling, and the fact that it has endured through so many difficulties is the proof of its power to create this affection in the hearts of countless men and women."

## Figures and Tables

**Figure f1:**
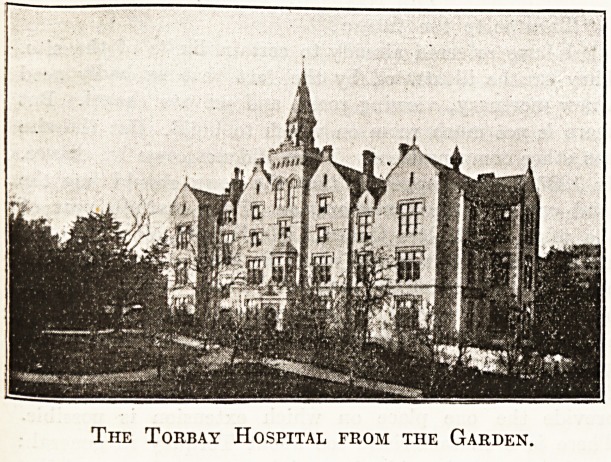


**Figure f2:**